# Treatment of liver fibrosis in hepatolenticular degeneration with traditional Chinese medicine: systematic review of meta-analysis, network pharmacology and molecular dynamics simulation

**DOI:** 10.3389/fmed.2023.1193132

**Published:** 2023-05-11

**Authors:** Xulong Yang, Tiancheng Wang, Yiping Tang, Yawen Shao, Yaqin Gao, Peng Wu

**Affiliations:** ^1^College of Integrated Traditional Chinese and Western Medicine, Anhui University of Chinese Medicine, Hefei, China; ^2^College of Nursing, Anhui University of Chinese Medicine, Hefei, China

**Keywords:** liver fibrosis, hepatolenticular degeneration, Wilson's disease, traditional Chinese medicine, meta-analysis, network pharmacology, molecular dynamics simulation

## Abstract

**Background:**

Traditional Chinese medicine (TCM) is widely used in the clinical treatment of hepatolenticular degeneration (HLD) and liver fibrosis (LF). In the present study, the curative effect was assessed using meta-analysis. The possible mechanism of TCM against LF in HLD was investigated using network pharmacology and molecular dynamics simulation.

**Methods:**

For literature collection, we searched several databases, including PubMed, Embase, Cochrane Library, Web of Science, Chinese National Knowledge Infrastructure (CNKI), VIP Database for Chinese Technical Periodicals (VIP) and Wan Fang database until February 2023, and the Review Manager 5.3 was used to analyze the data. Network pharmacology and molecular dynamics simulation were used to explore the mechanism of TCM in treating LF in HLD.

**Results:**

The results of the meta-analysis revealed that the addition of Chinese herbal medicine (CHM) in treating HLD resulted in a higher total clinical effective rate than western medicine alone [RR 1.25, 95% CI (1.09, 1.44), *p* = 0.002]. It not only has a better effect on liver protection [Alanine aminotransferase: SMD = −1.20, 95% CI (−1.70, −0.70), *p* < 0.00001; Aspartate aminotransferase: SMD = −1.41, 95% CI (−2.34, −0.49), *p* = 0.003; Total bilirubin: SMD = −1.70, 95% CI (−3.36, −0.03), *p* = 0.05] but also had an excellent therapeutic effect on LF through four indexes [Hyaluronic acid: SMD = −1.15, 95% CI (−1.76, −0.53), *p* = 0.0003; Procollagen peptide III: SMD = −0.72, 95% CI (−1.29, −0.15), *p* = 0.01; Collagen IV: SMD = −0.69, 95% CI (−1.21, −0.18), *p* = 0.008; Laminin: SMD = −0.47, 95% CI (−0.95, 0.01), *p* = 0.06]. Concurrently, the liver stiffness measurement decreased significantly [SMD = −1.06, 95% CI (−1.77, −0.36), *p* = 0.003]. The results of network pharmacological experiments and molecular dynamics simulation indicate that the three high-frequency TCMs (Rhei Radix Et Rhizoma-Coptidis Rhizoma-Curcumae Longae Rhizoma, DH-HL-JH) primarily act on the core targets (AKT1, SRC, and JUN) via the core components (rhein, quercetin, stigmasterol, and curcumin), regulate the signal pathway (PI3K-Akt, MAPK, EGFR, and VEGF signaling pathways), and play a role of anti-LF.

**Conclusion:**

Meta-analysis indicates that TCM is beneficial in treating HLD patients and improving LF. The present study successfully predicts the effective components and potential targets and pathways involved in treating LF for the three high-frequency CHMs of DH-HL-JH. The findings of the present study are hoped to provide some evidence support for clinical treatment.

**Systematic review registration:**

https://www.crd.york.ac.uk/PROSPERO, identifier: CRD42022302374.

## 1. Introduction

Hepatolenticular degeneration (HLD), also known as Wilson's disease (WD), is a rare chronic autosomal recessive disease that presents a hereditary copper metabolism disorder (1). A mutation in the copper-transporting P-type adenosine triphosphatase (ATP7B) gene on chromosome 13 causes WD. A functional defect in ATP7B can disrupt its corporation into apo ceruloplasmin and thus the formation of ceruloplasmin, resulting in excess intracellular copper accumulation in the liver and extrahepatic tissues (2, 3). WD has various clinical manifestations, including liver disease, neurological dysfunction or both (4). However, liver fibrosis (LF) is one of the major pathological changes in the liver of almost every WD patient (5). The global prevalence of WD is estimated to be 1/30,000 to 1/100,000 (1). WD can be fatal if left untreated. WD currently requires lifelong treatment, primarily through medical treatment (6). Despite a wide range of drug choices, patients with long-term or excessive drug use may experience significant adverse effects, such as nephrotoxicity, thrombocytopenia, complete aplastic anemia, myasthenia gravis, reversible iron cell anemia and hemosiderosis (7). Many studies have demonstrated that traditional Chinese medicine (TCM) in treating WD is effective and has few side effects, and can effectively expel copper and play the role of anti-WD LF.

However, under the guidance of the theory of TCM, individualized treatment is common in clinics. Although both patients are WD, the choice and compatibility of Chinese herbal medicine (CHM) differ. Simultaneously, some research reports have methodological defects, one-sided theoretical premises and a lack of experimental design, all of which reduce the effectiveness and universality of the research findings. In this systematic review, using an evidence-based research strategy, after a meta-analysis of clinically effective prescriptions in the treatment of WD, the three CHMs with the highest frequency of effective prescriptions were selected to investigate the mechanism of the treatment of liver fibers in WD using network pharmacology and molecular dynamics simulation. This is useful to determine future basic research direction and screen suitable active ingredients for clinical trials.

## 2. Methods

This systematic review with meta-analysis was registered with the number CRD42022302374 (https://www.crd.york.ac.uk/PROSPERO).

### 2.1. Search strategy

From inception to February 2023, we searched the following electronic databases: PubMed, Embase, Cochrane Library, Web of Science, Chinese National Knowledge Infrastructure (CNKI), VIP Database for Chinese Technical Periodicals (VIP) and Wan Fang database without language restrictions. The search strategy of PubMed was used, but it was modified to work with other English or Chinese databases. The PubMed search strategy was as follows:

#1

“Hepatolenticular Degeneration”[MeSH Terms] OR “hepatolenticular degeneration^*^”[Text Word] OR “Pseudosclerosis”[Text Word] OR “wilson disease^*^”[Text Word] OR “wilson s disease^*^”[Text Word] OR “wilsons disease^*^”[Text Word] OR “westphal strumpell syndrome^*^”[Text Word] OR “copper storage disease^*^”[Text Word] OR “progressive lenticular degeneration^*^”[Text Word] OR “neurohepatic degeneration^*^”[Text Word]

#2

“Liver Cirrhosis”[MeSH Terms] OR “hepatic cirrhosis”[Text Word] OR “cirrhosis hepatic”[Text Word] OR “cirrhosis liver”[Text Word] OR “fibrosis liver”[Text Word] OR “liver fibrosis”[Text Word]

#3

#1 AND #2

#4

“drugs, chinese herbal”[MeSH Terms] OR “Chinese Herbal Drugs”[Text Word] OR “Chinese Plant Extracts”[Text Word] OR “Herbal Medicine”[MeSH Terms] OR “medicine herbal”[Text Word] OR “Hawaiian Herbal Medicine”[Text Word] OR “la au lapa au”[Text Word] OR “Herbalism”[Text Word] OR “la au lapa au”[Text Word]

#5

(“controlled clinical trial”[Publication Type] OR “Controlled Clinical Trials as Topic”[MeSH Terms] OR “Random Allocation”[MeSH Terms] OR “Double-Blind Method”[MeSH Terms] OR “single-blind method”[MeSH Terms] OR “Control Groups”[MeSH Terms] OR “cross-over studies”[MeSH Terms] OR “random^*^”[Title/Abstract] OR “placebo”[Title/Abstract] OR “trial”[Title/Abstract] OR “groups”[Title/Abstract] OR “crossover”[Title/Abstract] OR “cross-over”[Title/Abstract]) NOT (“Animals”[MeSH Terms] NOT (“Humans”[MeSH Terms] AND “Animals”[MeSH Terms]))

#6

#3 AND #4 AND #5

For additional potential research, we also reviewed the list of references in relevant abstracts and original research articles.

### 2.2. Inclusion and exclusion criteria

#### 2.2.1. Inclusion criteria

1. Types of literature study: randomized controlled trials (RCTs).
2. Subjects: patients with clinically confirmed WD LF (as defined by the American Association of Hepatology Research Association clinical guidelines for WD (2008) (4), Chinese Association of Neurology guidelines for the diagnosis and treatment of HLD (2008) (8), or other diagnostic criteria with similar definitions), regardless of age, gender, disease course and severity.
3. Intervention: patients in the control group will receive routine Western medicine, while patients in the intervention group will receive TCM based on control Western medicine.
4. Outcome index: the main observation indexes include clinical effective rate (effective rate = effective number/total number, including recovery, significant effects, improvement, and patients without clinical symptoms before treatment) and liver manifestations (including four items of liver fiber serum, liver function, liver ultrasound, and liver stiffness measurement).

#### 2.2.2. Exclusion criteria

1. Trial group was only treated with TCM in the study design.
2. Non-randomized grouping; Randomized method obvious error.
3. *In vitro* or animal studies.
4. Case reports, reviews, meta-analyses or laboratory studies only.

To avoid the analysis of duplicate data, the latest study was included in the current study when two (or more) studies had the same cohort of patients.

### 2.3. Data extraction and quality assessment

Table 1 summarizes the characteristics of the ten trials. To extract data, standardized data extraction tables were used, which included patients, methods, interventions, and results. Parts of the study results were meta-analyzed by forest plot using Review Manager 5.3. Assess the risk of bias in included articles using the “bias risk assessment tool” provided by Cochrane.

**Table 1:** Characteristics of the included studies.

**References**	**Interventions (n) drug**		**Sample size**	**Sample and characteristics (male/female), age, duration**		**Course of treatment**	**Course of treatment outcomes**	**Intergroup differences**
	**Trial**	**Control**		**Trial**	**Control**			
Hong et al. (9)	GDP+DMPS	DMPS	100	31/19; (18.6 ± 4.7) y; (2.6 ± 0.8) y	26/24; (18.9 ± 6.8)y; (3.1 ± 1.2) y	8 w	1. Hepatic ultrasonography 2. Electrophoresis of serum protein 3. 24 h excretion of urinary copper	1. *p* < 0.01 2. *p* > 0.05 3. *p* < 0.05
Fang et al. (10)	GDT + DMPS	DMPS	80	29/11; 7–38 (25.4 ± 5.17) y; 3 m−11 y (21.3 ± 10.4) m	26/14; 6–35 (23.0 ± 6.15) y; 9 m−13 y (21.1 ± 12.3) m	62 d	1. Nervous system symptom score 2. 24 h excretion of urinary copper 3. Indicator of hepatic fibrosis	1. *P < * 0.05 2. *P < * 0.05 3. *P >* 0.05
Wang et al. (11)	GDL+GSH, DMPS	GSH, DMPS	78	23/16; (16.2 ± 9.9)y; (7.9 ± 5.3) y	22/17; (15.3 ± 9.1) y; (6.9 ± 5.2) y	4 w	1. TCM Syndrome Points 2. Efficacy of medical syndrome 3.Hcy level 4. Indicator of hepatic fibrosis	1.*P < * 0.05 2. p=0.165 3. *P < * 0.05 4. *P < * 0.05
Chen et al. (12)	GDL + GSH, PCA	GSH, PCA	29	8/6; 5–44 y (12.1 ± 9.1) y; 4 m−21.5 y (11.6 ± 8.7) y	7/8:6–43 y (11.7 ± 7.9)y; 5 m−22 y (10.2 ± 9.1) y	6 m	1. TCM Syndrome Points 2. Indicator of hepatic fibrosis 3. Liver function 4. Spleen length, thickness and portal vein width	1. *P < * 0.05 2. *P < * 0.05 3. *P < * 0.05 4. *P < * 0.05
Huang et al. (13)	GDL + GSH	GSH	60	16/14; (21.36 ± 4.45) y; (3.86 ± 2.38) y	14/16; (20.96 ± 4.36) y; (3.90 ± 2.12) y	32 d	1. Liver function 2. Indicator of hepatic fibrosis 3. Coagulation index 4. Child-Pugh score	1. *P < * 0.05 2. *P < * 0.05 3. *P < * 0.05 4. *P < * 0.05
Shi et al. (14)	SMTCM + DMPS	DMPS	90	32/13; 7–39 (23.6 ± 6.2) y; 4 m−13 y (18.9 ± 5.4) m	30/15; 8–36 (22.8 ± 5.9) y; 6 m−11 y (18.4 ± 6.1) m	50 d	1. Nervous system symptom score 2. Indicator of hepatic fibrosis 3. Liver function 4.24 h excretion of urinary copper	1. *P < * 0.01 2. *P >* 0.05 3. *P < * 0.05 4. *P < * 0.05
Chen (15)	GDL + DMSA	DMSA	61	20/11; 15–39 y (25.13 ± 7.77) y; 3–19 y (10.39 ± 4.50) y	17/13; 17–36 y (26.40 ± 6.18) y; 1–19 y (11.13 ± 4.88) y	48 w	1. TCM Syndrome Points 2. Liver function (ALT, AST) 3. Indicator of hepatic fibrosis 4. liver stiffness measurement 5. APRI Rating, FIB-4 index	1.*P < * 0.05 2.*P < * 0.05 3.HA, PC-III (*P < * 0.05); LN, C-IV (*P >* 0.05) 4.*P < * 0.05 5.*P >* 0.05
Ma et al. (16)	BSHXHR + DMPS	DMPS	42	22 / 20; 16–41 (27.8 ± 6.3) y		32 d	1. Indicator of hepatic fibrosis	1.*P < * 0.05
Yang (17)	GDFMD + DMSA	DMSA	58	12/16; 10–25 y (19.50 ± 4.75) y; (15.96 ± 7.24) m	16/14; 10–25 y (20.13 ± 5.35) y; (16.36 ± 8.64) m	24 w	1.Clinical efficacy 2. liver stiffness measurement 3. Liver function (ALT, AST) 4. Indica to Indicator of hepatic fibrosis 5. UWDRS Rating 6. Efficacy of TCM Syndrome	1. *P < * 0.05 2.*P < * 0.05 3. ALT, *P < * 0.01; AST, *P < * 0.05 4. HA,LN, C-IV (*P < * 0.01); PC-III (*P >* 0.05) 5. *P < * 0.05 6.*P < * 0.01
Zhang et al. (18)	GDL + DMPS	DMPS	76	22/18; 11–36 y (22.22 ± 7.72) y; 2–5 m (21.36 ± 10.87) m	18/18; 12–36 y (24.94 ± 7.49) y; 1–35 m (21.08 ± 11.04) m	48 d	1. liver stiffness measurement 2. APRI Rating 3. Indicator of hepatic fibrosis	1.*P < * 0.01 2.*P < * 0.05 3.HA, PC-III (*P < * 0.01): LN, C-IV (*P >* 0.05)

GDP, Gandou pian; GDT, Gandou tang; GDL, Gandouling tablet; SMTCM, Self-made traditional Chinese medicine decoction; BSHXHR, Bu Shen Huo Xue Hua Zhuo Recipe; GDFMD, Gan Dou Fu Mu decoction; DMPS, Sodium dimercaptosulphonate; GSH, Glutathione; PCA, Penicillamine; DMSA, Dimercaptosuccinic acid capsules; ALT, Alanine aminotransferase; AST, Aspartate aminotransferase; HA, Hyaluronic acid; C-IV, IV Collagen; PC-III, III procollagen peptide; LN, Laminin; y, year; m, month; d, day; w, week.

### 2.4. Network target analysis

#### 2.4.1. Database of network pharmacology

TCMSP database (https://tcmspw.com/index.php) was used to search for active ingredients and potential targets of “high-frequency TCM” (19). The active ingredients screening criteria are “OB ≥ 30%, DL ≥ 0.18” (20). The “Canonical SMILES” of the active ingredients were obtained from the PubChem database (https://pubchem.ncbi.nlm.nih.gov/) (21) and imported into the Swiss Target Prediction platform (http://www.swisstargetprediction.ch/) (22). The target of “probability > 0” was selected to supplement (23). Then, using the UniProt platform (https://www.uniprot.org/), standardize the protein name, limit the species to “homo sapiens,” and remove duplicate targets (24). The disease-related targets were identified using the keyword “LF” in the GeneCards database (https://www.genecards.org/) (25) and OMIM database (https://www.omim.org/) (26). The targets obtained in the GeneCards database were included in the “relevance score > 10” (11). The omicshare platform (https://www.omicshare.com) was used to obtain the intersection targets of “high-frequency TCM” and LF. Cytoscape3.8.2 Software created the “TCM-active ingredient-potential target” network (27).

### 2.5. Construction of PPI network and analysis of single-cell RNA sequencing data

The intersection target was imported into the STRING platform (https://www.string-db.org/) (28), and the protein classification was set to “homo sapiens” with a maximum confidence level of ≥0.7, masking the unconnected nodes in the network (29). Cytoscape 3.8.2 software was used to visualize the PPI network. Use cytoHubba (30) and MCODE (31) plug-ins to obtain the core targets under different algorithms (29). Because the LF process involves multiple cells, we evaluated the expression of core genes in these cells to determine whether they promote fibrosis by regulating the function of these cells. The PanglaoDB database (https://panglaodb.se/index.html) (32) was used to identify the cell lines with highly expressed core genes.

### 2.6. Enrichment analysis

The omicshare platform enriched the core genes by Gene Ontology (GO) and the Kyoto Encyclopedia of Genes and Genomes (KEGG). The primary use of GO functional analysis is to describe the functions of gene targets, including biological processes, cellular components and molecular functions. KEGG enrichment analysis can determine the signal pathway of intersection target enrichment. The Fisher test considered *p*-values < 0.05 and *Q*-values < 0.05 statistically significant.

### 2.7. Molecular dynamics simulation

The amber18 software package was used to perform the molecular dynamics (MD) simulation of the protein-small molecule complex. The protein uses the ff14SB force field parameter, whereas the small molecular ligand uses the gaff general force field parameter and calculates its AM1-BCC atomic charge using the ANTECHAMBER module. After loading the protein-small molecule complex into the tleap module, hydrogen atoms and antagonistic ions are automatically added to neutralize the charge. The TIP3P dominant water model is selected, and periodic boundary conditions are established. MD simulation workflow includes four steps: energy minimization, heating, balance, and production dynamics simulation. First, the energy of water molecules is minimized in 10,000 steps (including the 5,000-step steepest descent method and 5,000-step conjugate gradient method) by constraining the heavy atoms of proteins (and small molecules). The constraints are then released in 10,000 steps to optimize the energy of the entire system. After energy minimization, the system is slowly heated to 300 K at 50 ps time, and once heated, the system is balanced at 50 ps using the NPT ensemble. Finally, a 100 ns MD simulation is performed under an NPT ensemble with time step 2 fs. Every 20 ps, the trajectory data are saved, and the correlation analysis is performed using the CPPTRAJ module. The MMPBSA.py module calculated the binding free energy of ligand and protein.

## 3. Results

### 3.1. Study selection

**Figure 1:**
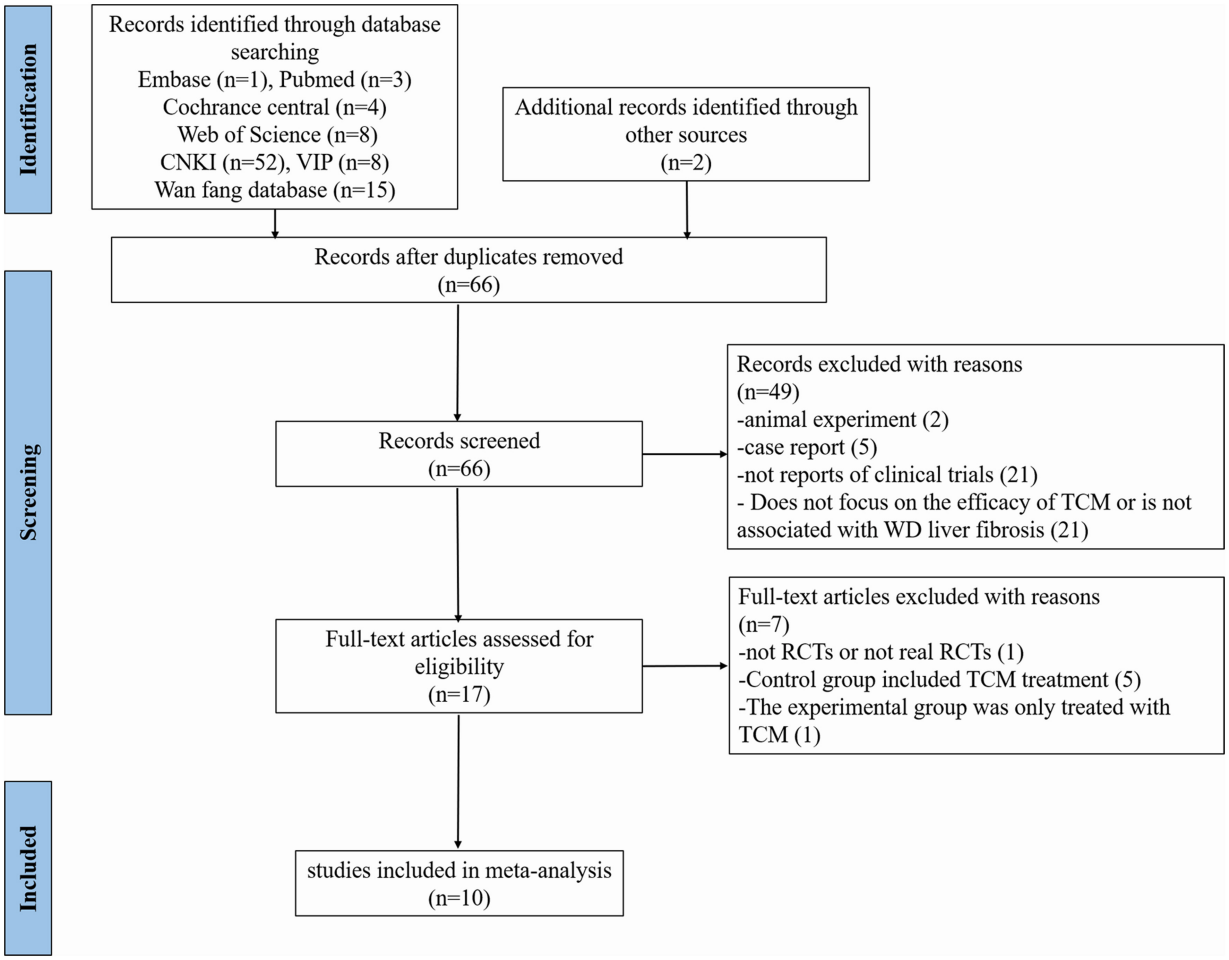
The screening flow-chat for the meta-analysis.

A preliminary search in the electronic database identified 91 studies. In addition, two potentially eligible records were identified through manual searches, of which 66 remained after the removal of duplicates. After screening titles and abstracts, 49 articles about non-clinical trials, animal trials, and case reports not focusing on the efficacy of TCM or not related to WD LF were excluded. Following a full-text evaluation of the remaining 17 articles, seven were excluded based on the research purpose and screening criteria (one non-randomized controlled trial, five control groups included TCM treatment, and one experimental group only TCM treatment). Finally, ten trials were identified (Figure 1).

#### 3.1.1. The characteristics of included studies

The included studies were all published in Chinese between 2000 and 2022. With 674 participants, all ten studies (9, 10, 12–18, 33) were RCTs of integrated traditional Chinese and Western medicine vs. Western medicine alone. The randomized controlled baseline was comparable; all reported clinical symptom improvement and varying degrees of curative effect on patients. Table 1 summarizes the characteristics of ten studies.

### 3.2. Risk of bias in included studies

**Figure 2:**
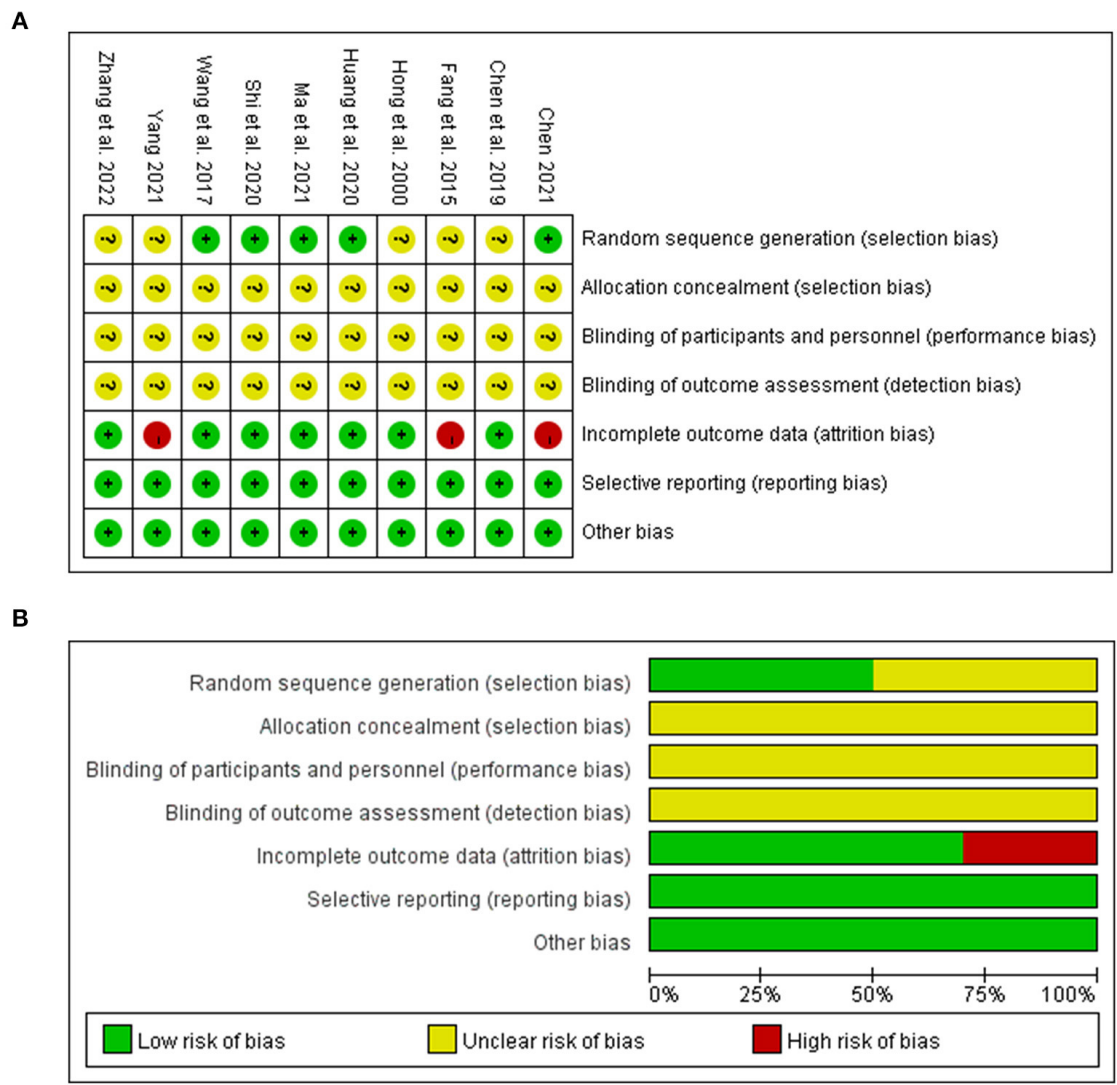
Risk of bias of the included studies. **(A)** Bias risk summary map. **(B)** The histogram for risk of bias.

The quality of the method is assessed using seven criteria in the Cochrane collaboration tool. All of the included studies indicated randomization. The method of randomization was reported in five studies (13–16, 33), which belonged to the low-risk category, while the other five articles did not specifically describe what type of randomization method was used, which belonged to the unclear-risk category. None of the ten studies addressed the risk of covert distribution and whether patients, researchers, and outcome reviewers were deceived. Two studies (15, 17) were withdrawn or lost follow-up, and the data were incomplete, placing them in the high-risk category, while the remaining eight had complete data and were classified as low-risk. All the literature has stated that the baseline is consistent and that no other bias is associated with low risk (Figure 2).

### 3.3. Results of meta-analysis

#### 3.3.1. The total clinical effective rate

**Figure 3:**
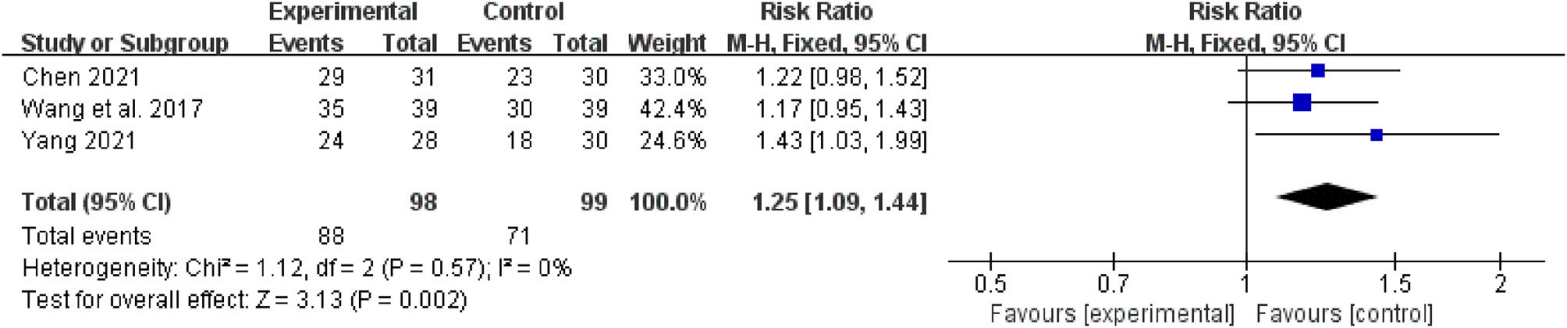
The forest plot: the total clinical effective rate of traditional Chinese medicine plus western medicine vs. western medicine.

Three studies with 197 participants reported specific clinical efficacy data. Heterogeneity test *p* = 0.57, *I*^2^ = 0%, using fixed effect model, total effect test *n* = 197, RR 1.25, 95% CI [1.09 to 1.44], Z = 3.31, *p* = 0.002 < 0.05; (Figure 3), indicating that TCM combined with western medicine has a higher total clinical effective rate than the control group.

#### 3.3.2. Liver function

**Figure 4:**
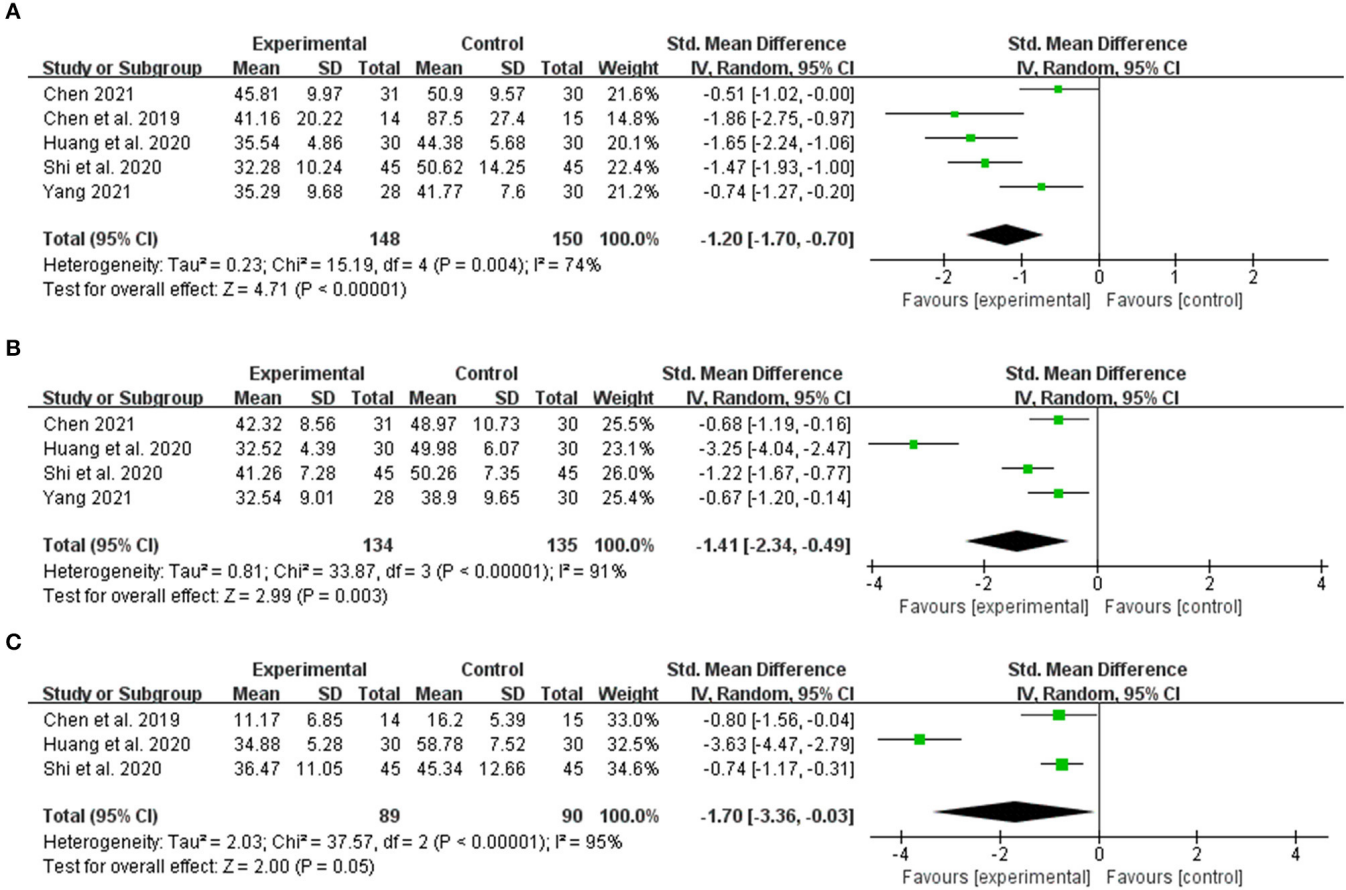
The forest plot. The liver function of traditional Chinese medicine plus western medicine vs. western medicine. **(A)** Alanine aminotransferase. **(B)** Aspartate aminotransferase. **(C)** Total bilirubin.

Five articles observed the index according to the alanine aminotransferase (ALT) index. Meta-analysis revealed that (n = 298, SMD = −1.20, 95% CI [−1.70 to −0.70]; Z = 4.71, *p* < 0.00001; heterogeneity: chi-square = 15.19, df = 4 (*p* = 0.004); *I*^2^ = 74%) (Figure 4A). Based on the aspartate aminotransferase (AST) index, four articles observed the index, and meta-analysis indicated (*n* = 269, SMD = −1.41, 95% CI [−2.34 to −0.49]; Z = 2.99, p = 0.003; heterogeneity: chi-square = 33.87, df = 3 (*p* < 0.00001); *I*^2^ = 91%) (Figure 4B). Based on the total bilirubin (TBIL) index, the meta-analysis revealed that (*n* = 179, SMD = −1.70, 95% CI [−3.36 to −0.03]; Z = 2.00, *p* = 0.05; heterogeneity: chi-square = 37.57, df = 2 (*p* < 0.00001); *I*^2^ = 95%) (Figure 4C). Within a specific range, the three liver function indexes in the integrated traditional Chinese and Western medicine treatment group are lower than in the simple western medicine group. This indicates that the integrated traditional Chinese and western medicine treatment has a better hepatoprotective effect.

#### 3.3.3. Indicator of LF

**Figure 5:**
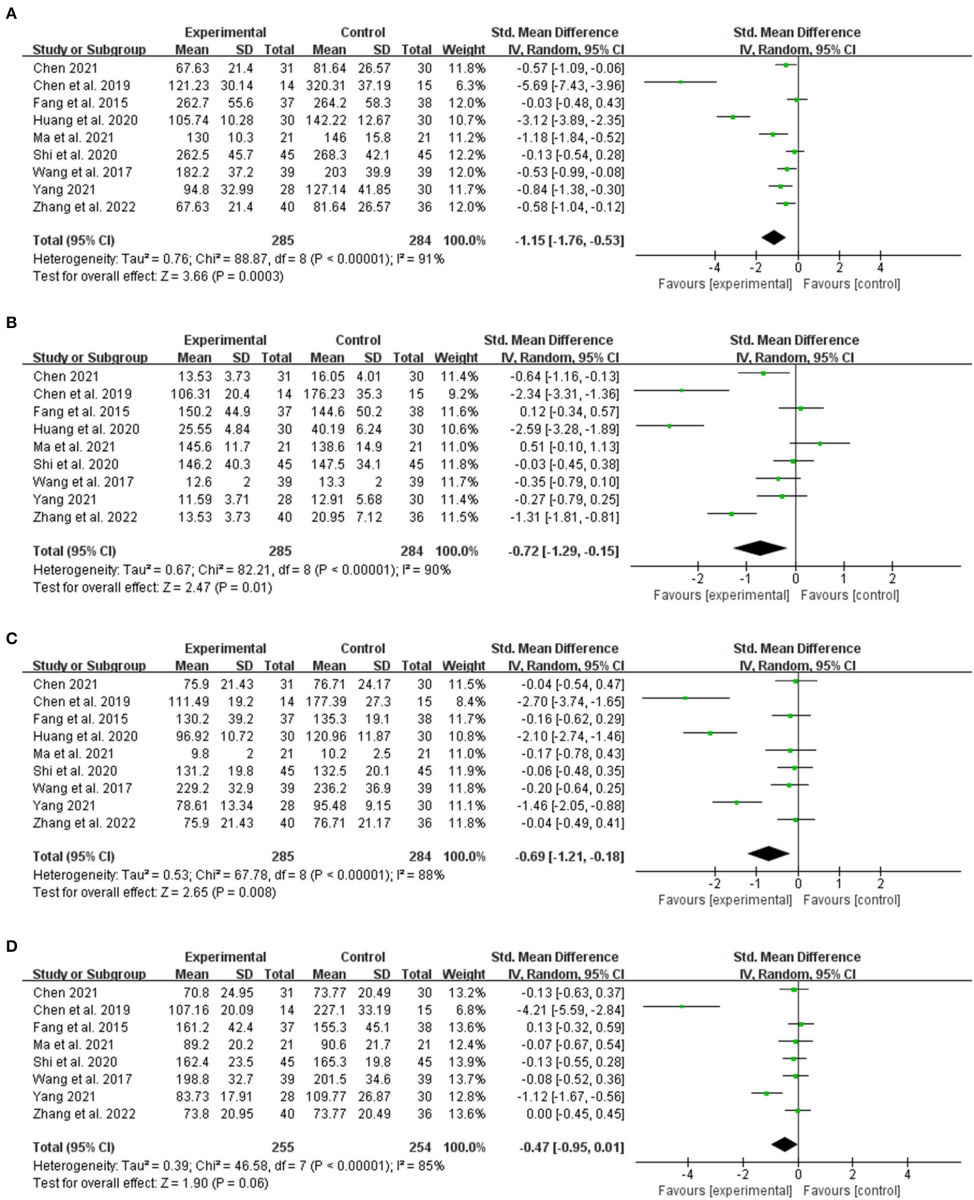
The forest plot. The liver fibrosis indices of traditional Chinese medicine plus western medicine vs. western medicine. **(A)** Hyaluronic Acid. **(B)** Procollagen peptide-III. **(C)** Collagen-IV. **(D)** Laminin.

The LF indices were observed in nine studies. The aggregate data revealed that CHM was more effective in reducing hyaluronic acid (HA) (*n* = 569, SMD = −1.15, 95% CI [−1.76 to −0.53]; Z = 3.66, *p* = 0.0003; heterogeneity: chi-square = 88.87, df = 8 (*p* < 0.00001); *I*^2^ = 91%) (Figure 5A), procollagen peptide III (PC-III) (*n* = 569, SMD = −0.72, 95% CI [−1.29 to −0.15]; Z = 2.47, *p* = 0.01; heterogeneity: chi-square = 82.21, df = 8 (*p* < 0.00001); *I*^2^ = 90%) (Figure 5B), and collagen IV (C-IV) (*n* = 569, SMD = −0.69, 95% CI [−1.21 to −0.18]; Z = 2.65 *p* = 0.008; heterogeneity: chi-square = 67.78, df = 8 (*P* < 0.00001); *I*^2^ = 88%) (Figure 5C) than the control group. Laminin (LN) index data were collected in eight studies. Meta-analysis indicated no significant difference between integrated traditional Chinese and Western medicine and Western medicine alone (*n* = 509, SMD = −0.47, 95% CI [−0.95 to 0.01]; Z = 1.90, p = 0.06; heterogeneity: chi-square = 46.58, df = 7 (*p* < 0.00001); *I*^2^ = 85%) (Figure 5D).

#### 3.3.4. Liver ultrasound

**Figure 6:**
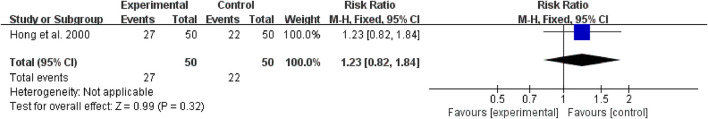
The forest plot. The Liver ultrasound of traditional Chinese medicine plus western medicine vs. western medicine.

Although only one study used liver ultrasound as a liver function index for observation and analysis, combining traditional Chinese and Western medicine significantly improves liver cirrhosis compared to Western medicine alone. The improvement rate of integrated traditional Chinese and Western medicine was 54.0% (27/50), while the simple Western medicine group improved at a rate of 44.0% (22/50), [RR 1.23, 95% CI 0.82 to 1.84] (Figure 6).

#### 3.3.5. Liver stiffness measurement

**Figure 7:**
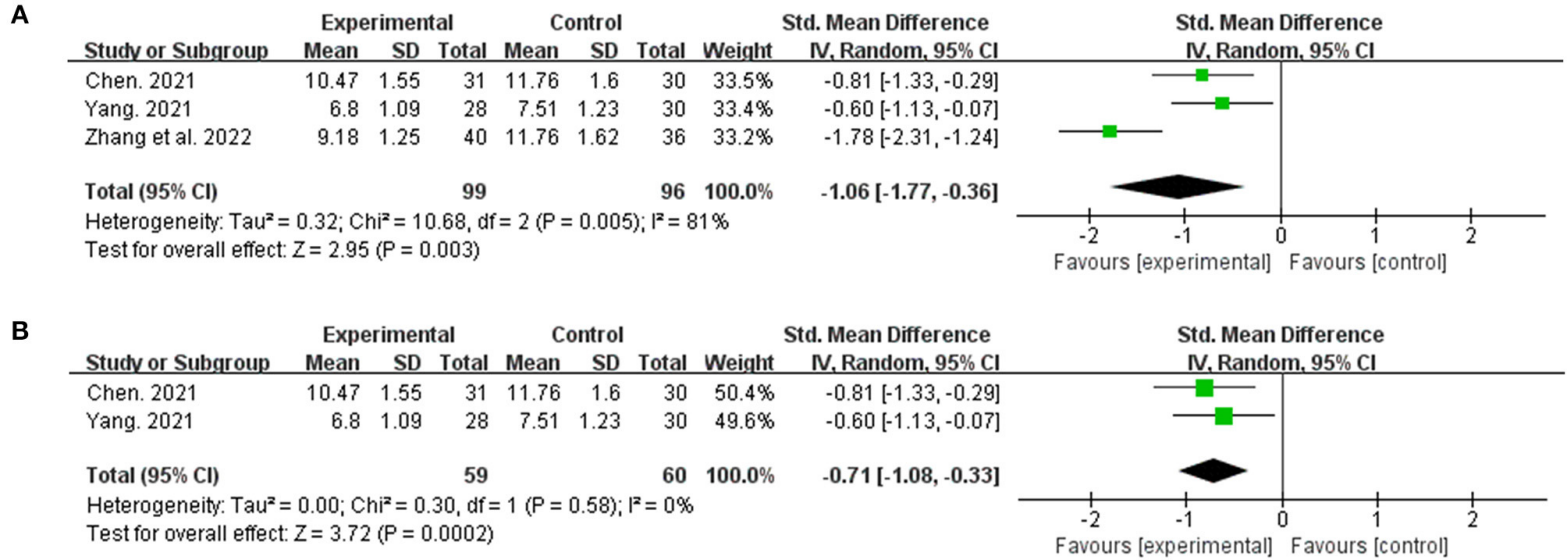
The forest plot. The liver stiffness measurement of traditional Chinese medicine plus western medicine vs. western medicine. **(A)** Forest plot of comparison before sensitivity analysis. **(B)** Forest plot of comparison after sensitivity analysis.

The LSM was observed in three studies. The combination of traditional Chinese and Western medicine can reduce LSM more effectively than Western medicine alone (*n* = 195, SMD = −1.06, 95% CI [−1.77 to −0.36]; Z = 2.95, *p* = 0.003) (Figure 7A). We analyzed its sensitivity by deleting the relevant studies one by one due to the high heterogeneity (*I*^2^ = 81%, *p* < 0.05) (Table 2). After excluding the study by Zhang et al. (18), the heterogeneity was reduced to 0% (p = 0.58) (Figure 7B).

**Table 2:** Results of sensitivity analysis of liver stiffness measurement.

**Eliminated literature**	**I^2^ (%)**	**Std. Mean Difference**	**95%CI**
Chen (15)	89%	−1.19	[-2.34,−0.04]
Yang (17)	84%	−1.29	[-2.24,−0.34]
Zhang et al. (18)	0%	−0.71	[-1.08,−0.33]

### 3.4. Treatment of WD with TCM

TCM treats WD by focusing on the concept, syndrome differentiation, and treatment. Several clinical and experimental studies revealed that TCM has an excellent effect on WD treatment. TCM believes that the cause of WD is a lack of congenital endowment and that the accumulation of copper toxin in the liver due to spleen deficiency is the leading cause of LF in WD. Numerous clinical data demonstrated that TCM could reduce the accumulation of copper in the body, protect and promote brain and liver function recovery, improve patient quality of life and prolong the life cycle (34, 35). Clinical data indicate that TCM can treat WD through quantitative compatibility of appropriate drugs via syndrome differentiation. Although TCM ingredient prescription has the advantages of definite clinical efficacy, ease of use, and low cost, it is also challenging to promote related research due to its multi-component, multi-level and multi-target characteristics. Therefore, in the present study, we chose to screen the CHM used in clinical trials to improve LF in WD and investigate the potential treatment mechanism of LF in WD using network pharmacology and MD simulation.

#### 3.4.1. High-Frequency TCM in effective TCM ingredient prescription for treatment of WD LF

Based on our review, we recorded and sorted the CHMs used in clinical trials that can effectively improve WD liver fibers (Table 3). The three most commonly used medicinal materials are Rhei Radix Et Rhizoma, Coptidis Rhizoma, and Curcumae Longae Rhizoma.

**Table 3:** Chinese herbal medicines used in clinical trials.

**Chinese Pinyin**	**Latin herb name**	**English herb name**	**Frequency (N/10) %**
Dahuang	Rhei Radix et Rhizoma	rhubarb root and rhizome	7 (70%)
Huanglian	Coptidis Rhizoma	Chinese Goldthread	7 (70%)
Jianghuang	Curcumae Longae Rhizoma	Turmeric	6 (60%)
Ezhu	Curcumae Rhizoma	Acruginous Turmeric Rhizome	5 (50%)
Jixueteng	Callerya reticulata (Benth.) Schot	Suberect Spatholobus Stem	4 (40%)
Danshen	Salviae Miltiorrhizae Radix et Rhizoma	Dan-Shen Root	4 (40%)
Yujin	Curcumae Radix	Aromatic Turmeric Root-tuber	2 (20%)
Zexie	Alisma orientale (Sam.) Juz.	Oriental Waterplantain Rhizome	2 (20%)
Sanqi	Panax notoginseng	Radix Notoginseng	2 (20%)
Jinqiancao	Lysimachiae Herba	Christina Loosestrife Herb	1 (10%)
Chaihu	Bupleuri Radix	Chinese Thorawax Root	1 (10%)
Bixie	Rhizoma Dioscoreae Collettii	Sevenlobed YamRhizome	1 (10%)
Banzhilian	ScutellariabarbataD. Don	Scutellaria barbata	1 (10%)
Chuanxinlian	Andrographis Herba	Common Andrographis Herb	1 (10%)
Yuxingcao	Houttyniae Herba	Heartleaf Houttuynia Herb	1 (10%)
Heshouwu	Polygoni Multiflori Radix	Tuber Fleeceflower Root	1 (10%)
Gouqizi	Lycii Fructus	Babury Wolfberry Fruit	1 (10%)
Baishao	Paeoniae Radix Alba	White Peony Root	1 (10%)
Fuling	Poria	Indian Buead	1 (10%)

### 3.5. The potential mechanism of CHM in the treatment of WD LF

#### 3.5.1. Screening core ingredients

**Figure 8:**
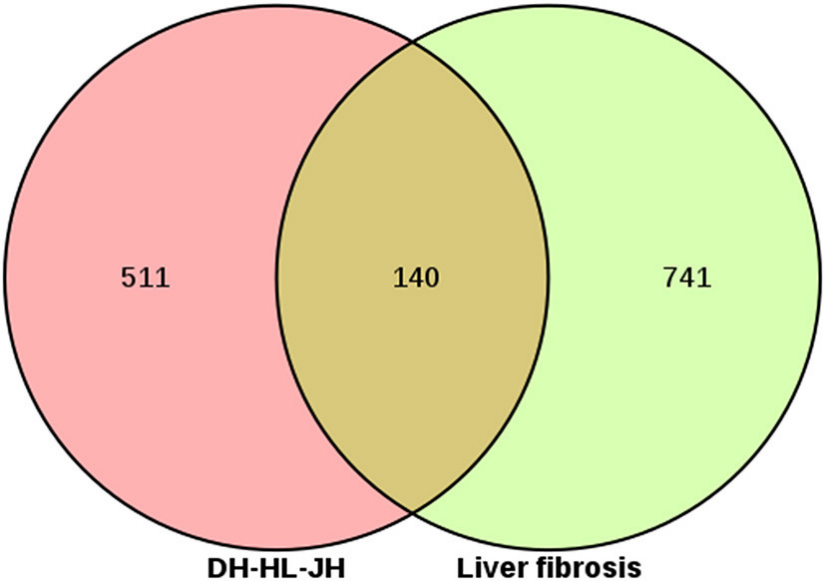
Venn diagram of Rhei Radix Et Rhizoma-Coptidis Rhizoma-Curcumae Longae Rhizoma (DH-HL-JH) against liver fibrosis.

**Figure 9:**
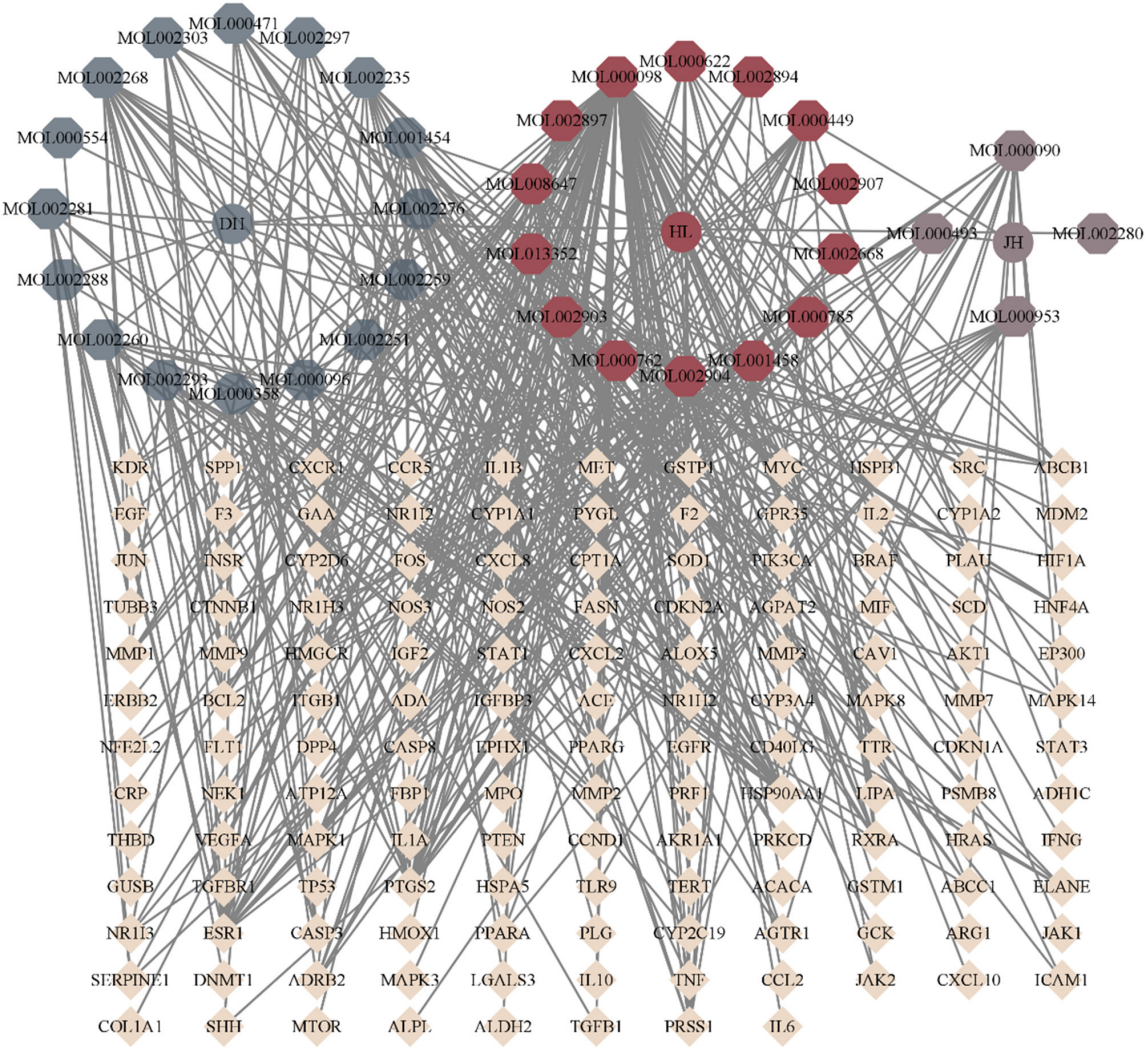
Target network of Rhei Radix Et Rhizoma-Coptidis Rhizoma-Curcumae Longae Rhizoma (DH-HL-JH) against liver fibrosis.

TCMSP database yielded 16 active ingredients of Rhei Radix Et Rhizoma, 14 Coptidis Rhizoma, and two species of Curcumae Longae Rhizoma. Simultaneously, a literature search reveals that curcumin can effectively inhibit the occurrence and development of LF. Therefore, it is supplemented. Table 4 presents the details of the active ingredients. By combining the TCMSP and SwissTargetPrediction database, 651 “DH-HL-JH” targets were identified. The OMIM database yielded 151 LF-related targets, while the GeneCards database identified 772 targets. After removing duplicate targets and standardizing gene names, 881 LF targets were obtained. The Venn diagram shows 140 intersection targets (Figure 8). “The node Degree value of DH-HL-JH” anti-fibrosis target network reveals that rhein, eupatin, quercetin and Berlambine, and Stigmasterol and Curcumin are the main components of Rhei Radix Et Rhizoma, Coptidis Rhizoma and Curcumae Longae Rhizoma, respectively (Figure 9).

**Table 4:** Details of the active components of 34 species of Rhei Radix Et Rhizoma-Coptidis Rhizoma-Curcumae Longae Rhizoma.

**Mol ID**	**Molecule name**	**OB (%)**	**DL**	**Source**
MOL002235	EUPATIN	50.8	0.41	Rhei Radix Et Rhizoma
MOL002251	Mutatochrome	48.64	0.61	Rhei Radix Et Rhizoma
MOL002259	Physciondiglucoside	41.65	0.63	Rhei Radix Et Rhizoma
MOL002260	Procyanidin B-5,3'-O-gallate	31.99	0.32	Rhei Radix Et Rhizoma
MOL002268	Rhein	47.07	0.28	Rhei Radix Et Rhizoma
MOL002276	Sennoside E_qt	50.69	0.61	Rhei Radix Et Rhizoma
MOL002280	Torachrysone-8-O-beta-D- (6'-oxayl)-glucoside	43.02	0.74	Rhei Radix Et Rhizoma
MOL002281	Toralactone	46.46	0.24	Rhei Radix Et Rhizoma
MOL002288	Emodin-1-O-beta-D-glucopyranoside	44.81	0.8	Rhei Radix Et Rhizoma
MOL002293	Sennoside D_qt	61.06	0.61	Rhei Radix Et Rhizoma
MOL002297	Daucosterol_qt	35.89	0.7	Rhei Radix Et Rhizoma
MOL002303	Palmidin A	32.45	0.65	Rhei Radix Et Rhizoma
MOL000358	Beta-sitosterol	36.91	0.75	Rhei Radix Et Rhizoma
MOL000471	Aloe-emodin	83.38	0.24	Rhei Radix Et Rhizoma
MOL000554	Gallicacid-3-O- (6'-O-galloyl)-glucoside	30.25	0.67	Rhei Radix Et Rhizoma
MOL000096	(-)-Catechin	49.68	0.24	Rhei Radix Et Rhizoma
MOL001454	Berberine	36.86	0.78	Coptidis Rhizoma
MOL013352	Obacunone	43.29	0.77	Coptidis Rhizoma
MOL002894	Berberrubine	35.74	0.73	Coptidis Rhizoma
MOL002897	Epiberberine	43.09	0.78	Coptidis Rhizoma
MOL002903	(R)-canadine	55.37	0.77	Coptidis Rhizoma
MOL002904	Berlambine	36.68	0.82	Coptidis Rhizoma
MOL002907	Corchoroside A_qt	104.95	0.78	Coptidis Rhizoma
MOL000622	Magnograndiolide	63.71	0.19	Coptidis Rhizoma
MOL000762	Palmidin A	35.36	0.65	Coptidis Rhizoma
MOL000785	Palmatine	64.6	0.65	Coptidis Rhizoma
MOL000098	Quercetin	46.43	0.28	Coptidis Rhizoma
MOL001458	Coptisine	30.67	0.86	Coptidis Rhizoma
MOL002668	Worenine	45.83	0.87	Coptidis Rhizoma
MOL008647	Moupinamide	86.71	0.26	Coptidis Rhizoma
MOL000449	Stigmasterol	43.83	0.76	Curcumae Longae Rhizoma
MOL000493	Campesterol	37.58	0.71	Curcumae Longae Rhizoma
MOL000953	Clr	37.87	0.68	Curcumae Longae Rhizoma
MOL000090	Curcumin	5.15	0.41	Curcumae Longae Rhizoma

#### 3.5.2. Screening core genes

**Figure 10:**
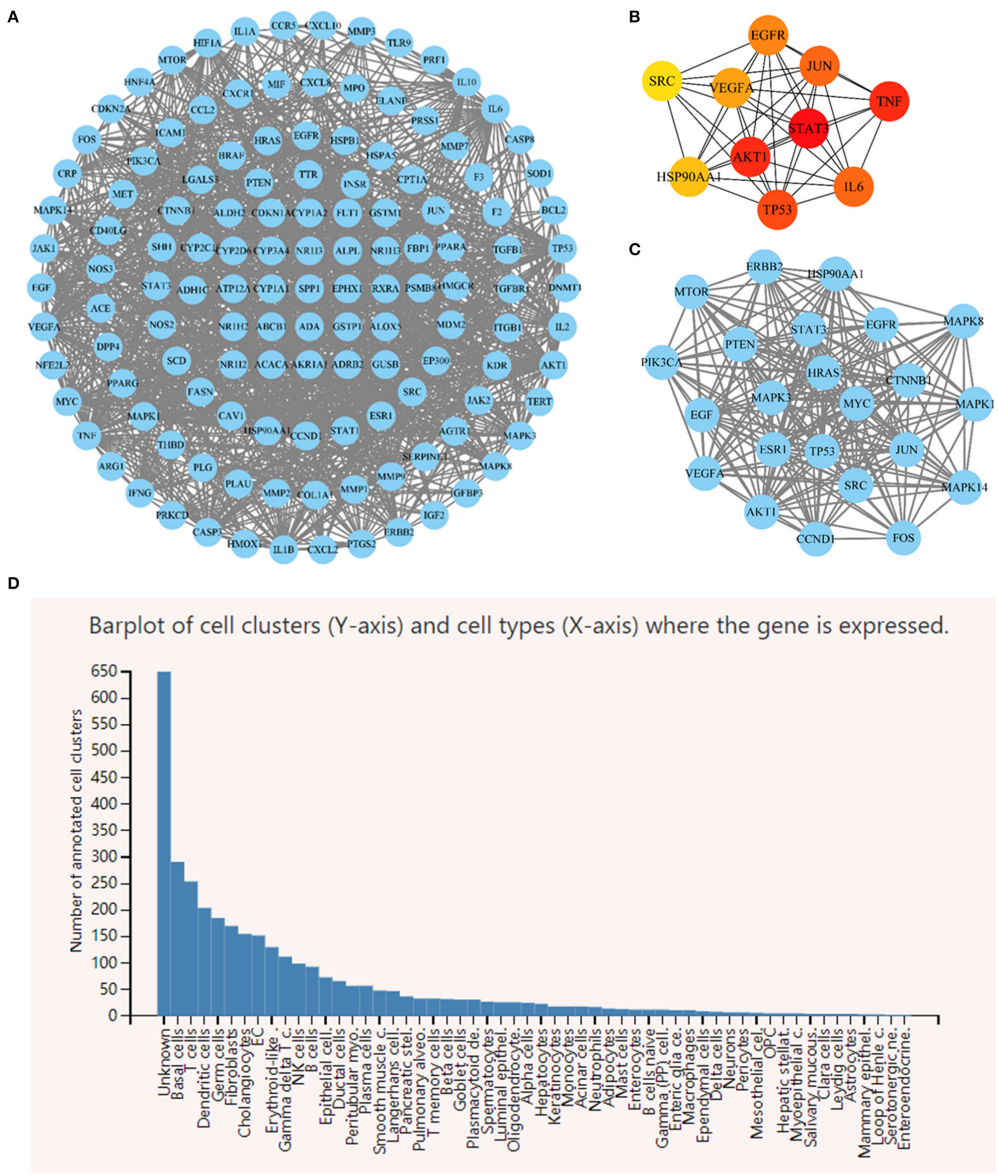
**(A)** Rhei Radix Et Rhizoma-Coptidis Rhizoma-Curcumae Longae Rhizoma (DH-HL-JH) pharmacodynamic composition-LF target PPI network. **(B)** Top 10 hub target genes ranked by node degree. **(C)** The most significant modules analyzed by the MCODE plugin. **(D)** Single cell RNA sequencing analysis of 8 core genes.

The PPI network included 131 nodes and 1,212 edges (Figure 10A). Nine targets were excluded from the PPI network due to a lack of interaction with other targets. The cytoHubba plug-in was used to determine the degree of connectivity for each target (Figure 10B). STAT3, VEGFA, EGFR, HSP90AA1, AKT1, TP53, SRC, JUN, IL6, and TNF were the top ten hub target genes according to node degree. These target genes could play an important role in the network. The MCODE plug-in was used to identify the most significant modules, with an MCODE score of 17.238 and containing 23 nodes and 214 edges. MAPK14, FOS, MAPK3, MAPK8, EGF, AKT1, ESR1, CCND1, SRC, CTNNB1, EGFR, HSP90AA1, PTEN, TP53, MAPK1, MTOR, PIK3CA, JUN, HRAS, ERBB2, STAT3, MYC, and VEGFA were the targets in the MOCDE score (Figure 10C). The findings revealed that the most important targets under various algorithms were STAT3, VEGFA, EGFR, HSP90AA1, AKT1, TP53, SRC, and JUN. Panglao DB database analysis identified that eight core genes were primarily expressed in fibroblasts, HSCs and other cell lines associated with the LF progression (Figure 10D).

**Figure 11:**
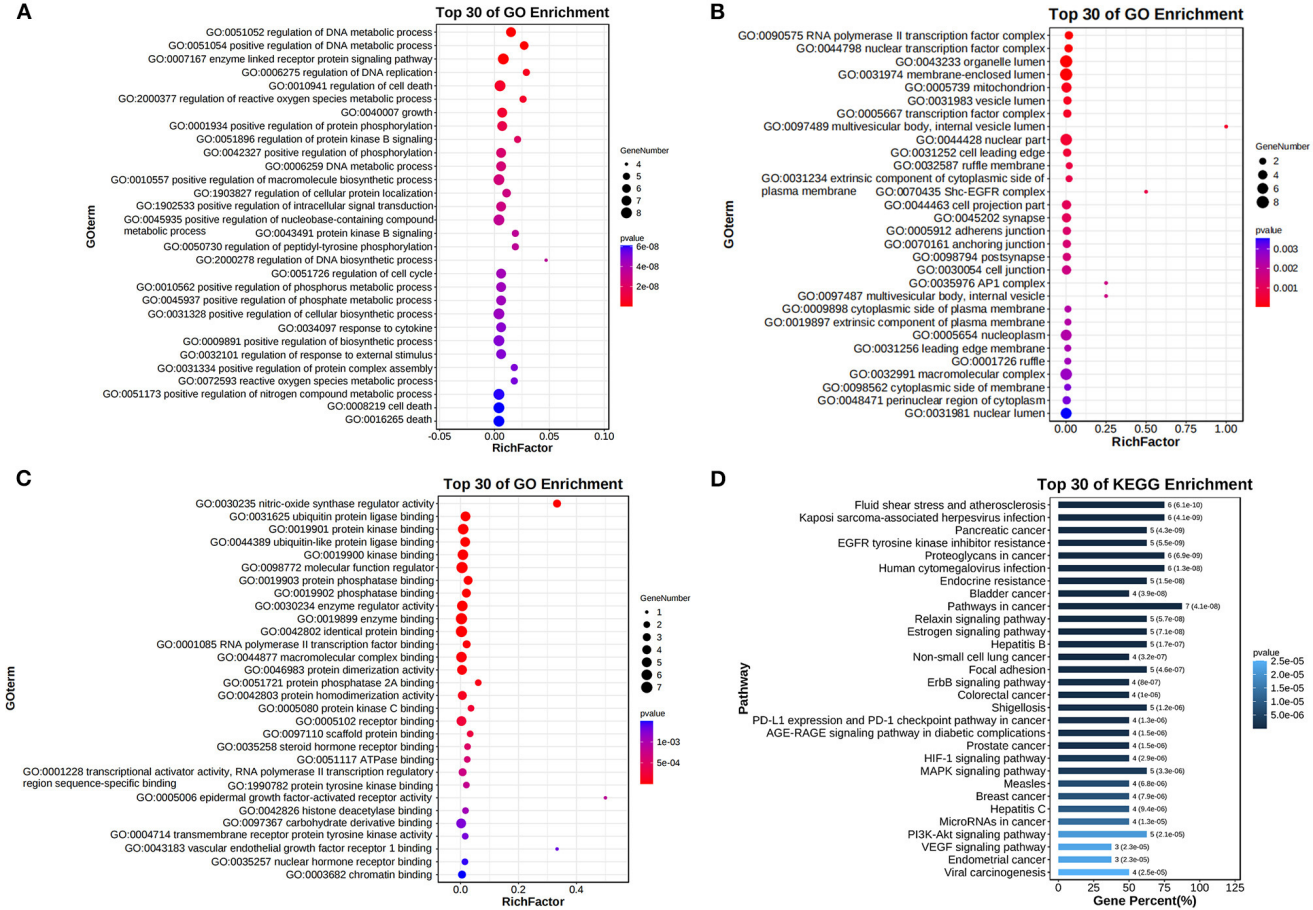
Gene Ontology enrichment analysis and Kyoto Encyclopedia of Genes and Genomes (KEGG) pathway analysis. **(A)** Top 30 items of BP enrichment. **(B)** Top 30 items of CC enrichment. **(C)** Top 30 items of MF enrichment. **(D)** Top 30 items of KEGG pathway analysis.

We intercepted the first 30 terms, from smallest to largest, based on the adjusted *p*-value. The findings of the biological process analysis revealed that the intersection targets were primarily enriched in the regulation of DNA replication, cell death, reactive oxygen species metabolic process, growth, and positive regulation of phosphorylation, as shown in Figure 11A. The results of cell composition demonstrated that the intersection targets were mostly enriched in organelle lumen, mitochondrion, multivesicular body, nuclear part, and cell leading edge, as presented in Figures 11B, C illustrates that molecular functions primarily include kinase binding, molecular function regulator, phosphatase binding, enzyme regulator activity, and enzyme binding.

We intercepted the top 30 KEGG pathways from smallest to largest based on the *p*-value. The analysis revealed that these targets were mostly enriched in the PI3K-Akt, EGFR, MAPK, and VEGF signaling pathways, as presented in Figure 11D.

#### 3.5.3. Molecular docking result

**Figure 12:**
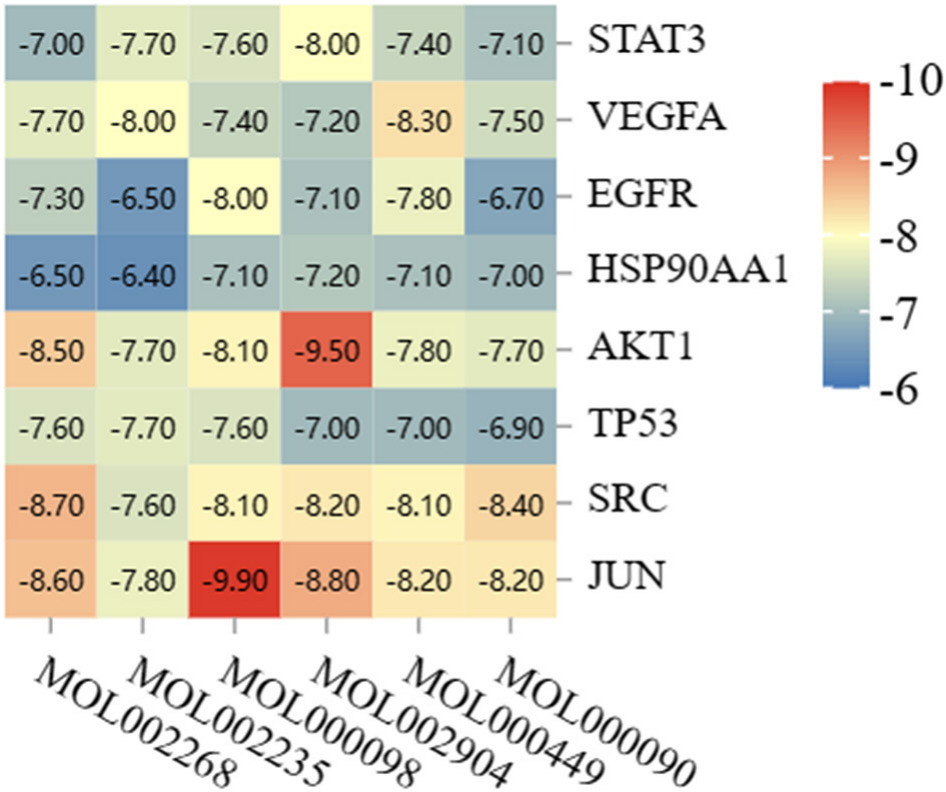
Heat map of molecular docking results.

**Figure 13:**
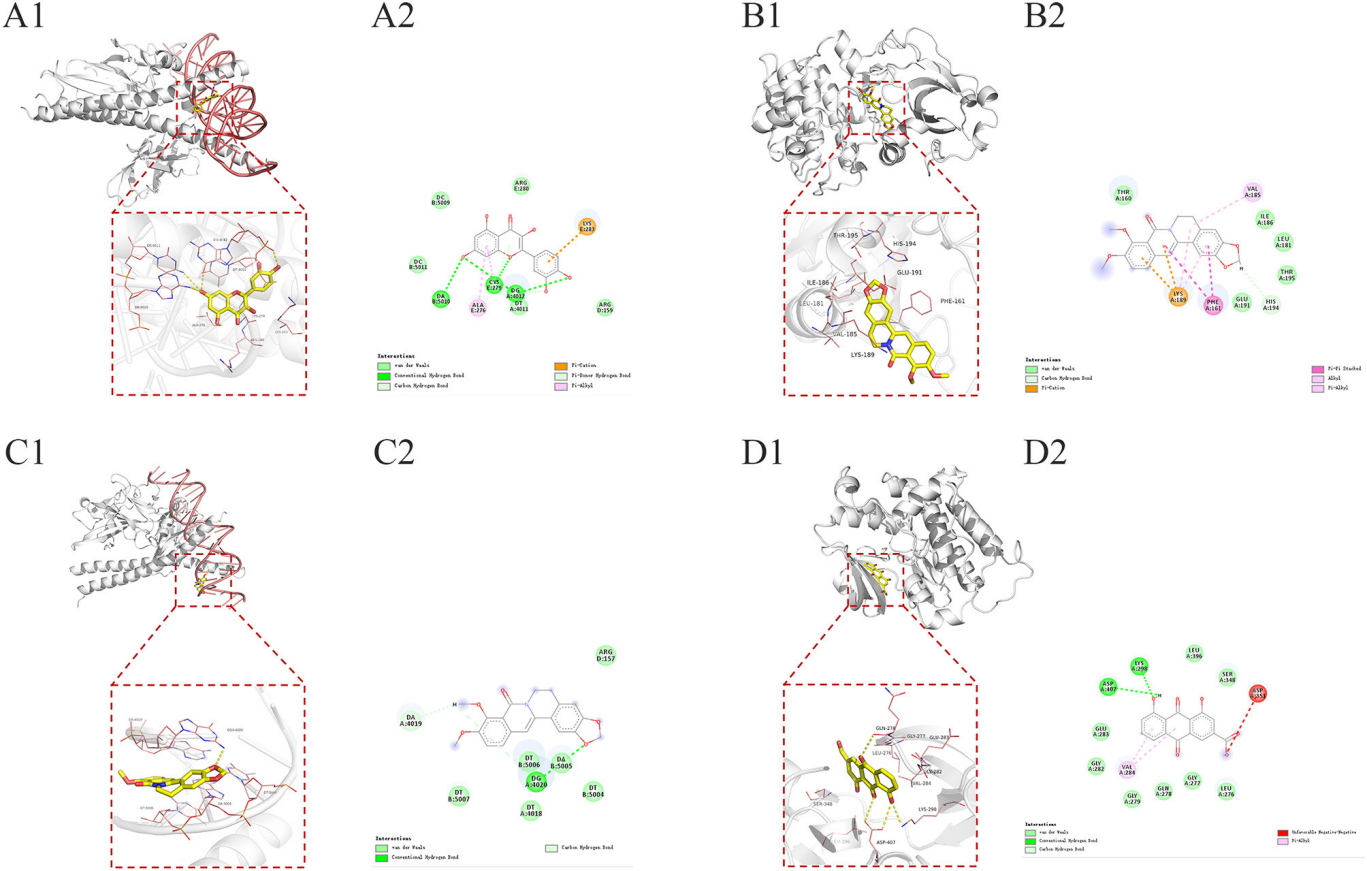
Docking patterns of key targets and specific active compounds. Quercetin-JUN **(A1)**, Berlambine-AKT1 **(B1)**, Berlambine-JUN **(C1)**, and Rhein-SCR **(D1)**. **(A2)**, **(B2)**, **(C2)**, and **(D2)**: Two dimensional patterns of bond, respectively.

Eight core targets and six key ingredients were docked molecularly. Rhein, eupatin, quercetin, berlambine, stigmasterol, and curcumin all had high binding energies to the core targets, with quercetin and JUN having the highest binding energy of −9.9 kJ/mol (Figure 12). The molecular docking results revealed that the active chemical ingredients of DH-HL-JH had low conformational energy with the core proteins and had high structural stability and binding activity (Figure 13).

#### 3.5.4. MD simulation

**Figure 14:**
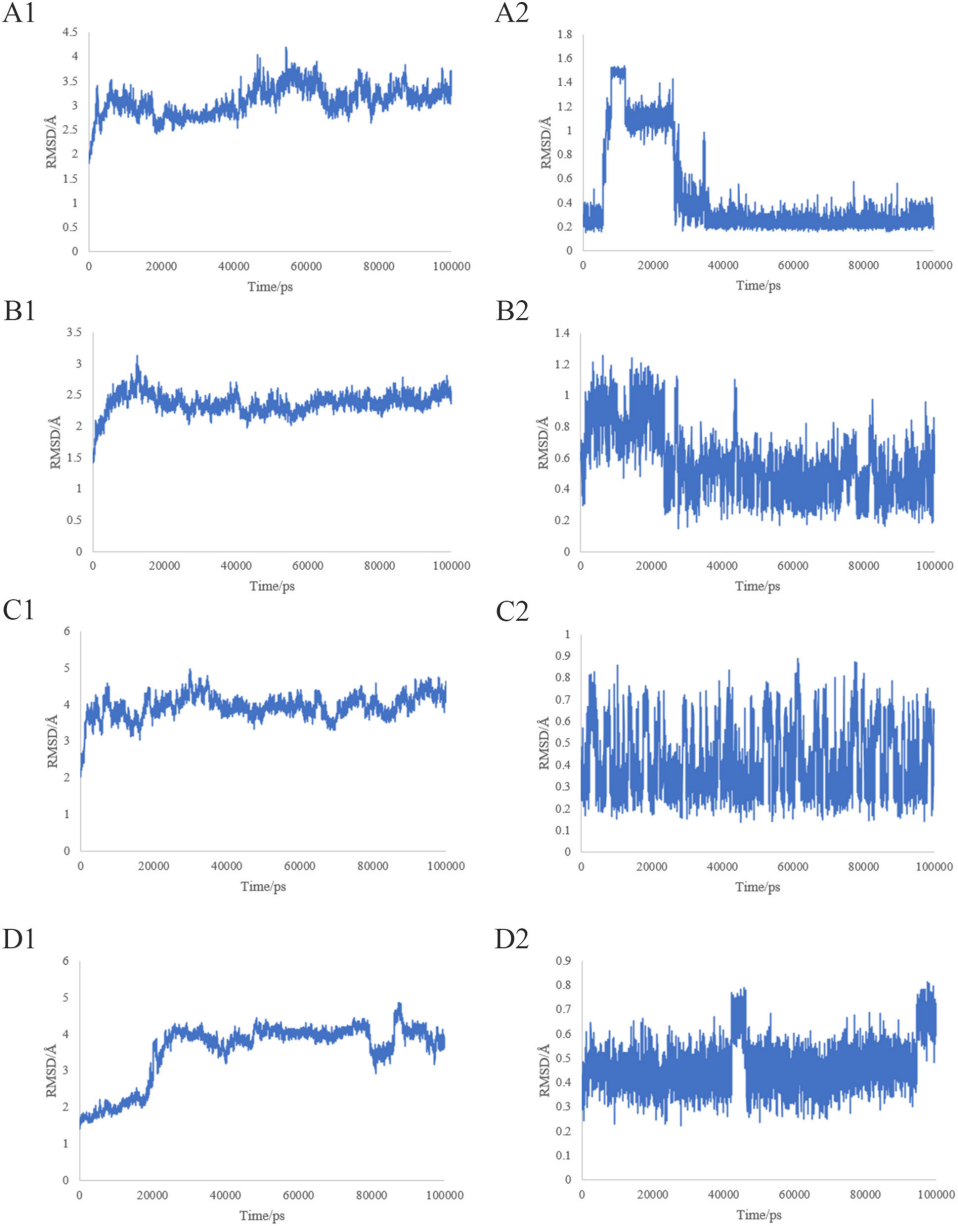
Root-mean-square deviation (RMSD) plots during of molecular dynamics simulation. **(A1–D1)** The RMSD of the protein in the complex. **(A2–D2)** The RMSD of the small molecule in the complex.

MD simulations reveal information about the stability of protein-ligand complexes. The present study performed 100 ns MD simulations of JUN-quercetin, AKT1-Berlambine, JUN-Berlambine and SCR-Rhein based on the docking results to evaluate the molecular motion, trajectory, structural characteristics, binding potential and conformational changes. The root mean square deviation (RMSD) curve represents the fluctuation of the protein conformation. The RMSD of the protein in the complex is on the left (Figures 14A1–D1), and the small molecule RMSD is on the right side (Figures 14A2–D2). Figure 14A presents that during the entire simulation process, the protein RMSD fluctuated slightly and stabilized around 3.5 Å. In contrast, the small molecule RMSD fluctuated somewhat, particularly in the pre-30 ns, but then abruptly decreased and stabilized at around 0.3 Å. Figure 14B reveals that the fluctuation of protein RMSD is slight throughout the simulation process, stabilizing around 2.5 Å. In contrast, the fluctuation of small molecule RMSD is smaller, essentially stable around 0.60 Å. Figure 14C demonstrated that the protein RMSD fluctuated slightly during the simulation and stabilized around 4 Å. In contrast, the small molecule RMSD fluctuated slightly, generally in the 0.2 to 0.8 Å. It is evident from Figure 14D that the pre-25 ns protein RMSD fluctuates significantly and stabilizes around 4 Å after 25 ns. In contrast, the small molecule RMSD fluctuates less, and the pre-90 ns usually is stable around 0.5 Å. Finally, 10 ns fluctuates to some extent before stabilizing at about 0.65 Å.

## 4. Discussion

In the present study, meta-analysis was used to assess the efficacy of traditional CHM in treating WD. We identified that interventional therapy with TCM brought positive results with few adverse reactions in ten clinical trials. Evidence of a more effective combination of traditional Chinese and Western medicine is emerging, which may assist in recommending clinical treatment. However, more high-quality trials are required to confirm this hypothesis. Simultaneously, based on the effectiveness of TCM in the clinical treatment of WD, we investigated its efficacy in treating WD LF. Nine trials using four indicators of LF reveal that integrated traditional Chinese and Western medicine can more effectively improve WD LF. Because clinical manifestations of LF lack specificity (36), the observation of the efficacy of clinical LF may also be combined with more and better indicators. In the last 2 years, we have identified that the observation of LSM value has been added to some literature. Concurrently, the analysis results confirm that treatment with integrated traditional Chinese and Western medicine has a better effect on WD LF improvement. Therefore, we can speculate that the addition of CHM contributed to the occurrence of this phenomenon. Rhei Radix Et Rhizoma, Coptidis Rhizoma, Curcumae Longae Rhizoma and other high-frequency CHM are more effective in treating and improving liver function in WD patients.

Based on the standard of “OB ≥ 30% and DL ≥ 0.18,” we searched the TCMSP database and identified 34 “DH-HL-JH” active ingredients via a literature supplement. Then, using the OMIM and GeneCards databases, we identified 881 LF disease targets. A total of 140 potential “DH-HL-JH”-LF targets were identified. According to the degree value of the nodes of the “DH-HL-JH” anti-LF target network, the main ingredients of Rhei Radix Rt Rhizome, Coptidis Rhizome, and Curcumae Longae Rhizome are rhein and eupatin, quercetin and berlambine, and stigmasterol and curcumin.

The liver of WD patients can exhibit various injury patterns, with LF being one of the pathological processes. LF is part of the tissue repair response to chronic liver injury (37). Whatever causes chronic liver injury, hepatic stellate cells (HSCs) are the main effector cells of LF, and HSC activation is a central link in the LF progression (38). Rhein is a lipophilic anthraquinone with a tricyclic aromatic anthraquinone structure (39). Rhein has been shown to significantly reduce the activity of ALT, the concentration of HA and PC-III, and the malondialdehyde (MDA) levels in the liver of carbon tetrachloride (CCl_4_) / ethanol injured rats, as well as the expression of α-SMA and TGF-β1. Rhein can prevent LF by inhibiting TGF-β1 and activating HSCs (40). Li et al. (39) demonstrated that rhein could effectively reduce the protein and mRNA expression of α-SMA and metalloproteinase-1 (TIMP-1), biomarkers of HSC activation in LF, and delay the progression of LF. Quercetin is a flavonoid in many plants and acts as a natural polar auxin transport inhibitor (41). The development of preclinical evidence involving 254 animals revealed that quercetin significantly affected LF, particularly over 4–8 weeks and at a 5–200 mg dose. It is suggested that quercetin has excellent potential in treating LF (42).

Curcumin, a diarylheptane derivative found in Curcumae Longae Rhizoma, is a powerful fat-soluble antioxidant (43). Curcumin inhibits hepatocyte epithelial-mesenchymal transition to alleviate LF by regulating oxidative stress and autophagy (44). Curcumin can prevent LF by inducing apoptosis and inhibiting HSC activation and VEGF expression (30, 45). Stigmasterol is an unsaturated phytosterol in the class of tetracyclic triterpenoid steroids. Studies indicate it can trigger PI3K/Akt, Akt/mTOR, JAK/STAT, VEGFR-2 and other signal pathways (46). Nitha et al. described that stigmasterol protects against CCl_4_-induced LF (47). The active ingredients in “DH-HL-JH” have well-established therapeutic effects in LF.

Using cytoHubba and MCODE plug-in in Cytoscape software, eight core targets are obtained using different algorithms. The target AKT1 is an AKT kinase subtype. Reyes-Gordillo et al. demonstrate that AKT1 participates in ethanol-and lipopolysaccharide-mediated LF and inhibits hepatocytes and HSC fibrogenesis and proliferation, which plays a unique role in the fibrosis process (48). SRC kinase is a non-receptor membrane tyrosine kinase. Seo et al. (49) revealed that SRC is up-regulated during HSC and LF activation. SRC inhibition can reduce LF by preventing HSC activation, increasing autophagy flux, and reducing connective tissue growth factor. JUN protein is an important component of the AP-1 dimer. The delicate balance of AP-1 expression and activity will respond to clues from the activated HSC microenvironment. JUN is involved in HSC activation and fibrosis (50).

GO function analysis revealed that various biological functions, including DNA metabolic process, cell death, reactive oxygen species metabolic process and signal transduction, were linked to the role of “DH-HL-JH” in LF. The results of the KEGG pathway analysis indicated that multiple pathways, including PI3K-Akt, MAPK, VEGF signaling pathways and EGFR tyrosine kinase inhibitor resistance, were linked to the role of “DH-HL-JH” in LF. Sun et al. (51) demonstrated that membrane receptors activate the pathways related to rhein targets, which activate MAPK and PI3K-AKT parallel signaling pathways, affecting several downstream pathways and ultimately regulating cell cycle and apoptosis. Wu et al. (52) indicated that quercetin could increase PI3K and p-Akt expression in fibrosis models and prevent LF by activating the PI3K-Akt signal pathway to reduce autophagy and inhibit HSC activation. A study on the therapeutic effect of oxidized berberine on obese rats with non-alcoholic fatty liver disease revealed that it significantly inhibited the abnormal phosphorylation of IRS-1, up-regulated the expression of downstream proteins and phosphorylation (PI3K, p-Akt/Akt and p-GSK-3 β / GSK-3 β), and improved liver insulin signal transduction (53). The imbalance of the MAPK signal pathway is the primary cause of inflammation, and P38 MAPK signaling is critical for LF development (54). Related studies have shown that regulating the composition of intestinal microorganisms can reduce the phosphorylation of MAPK and AKT proteins and improve inflammatory bowel disease via PI3K and MAPK signaling pathways, which can inhibit pathogenic bacteria and their metabolites in the liver, thereby reducing LF (55).

Finally, docking was performed on six core ingredients and eight key target proteins. The binding affinities of the docking results ranged from −6.4 to −9.9 kcal/mol, indicating that all targets may have good docking ability with active ingredients. The docking results revealed that the four JUN-quercetin, AKT1-berlambine, JUN-berlambine, and SRC-rhein groups had a low binding affinity. The interaction of the four groups mentioned above was studied further by MD simulation. The visualization of the RMSD curve in the JUN-quercetin group revealed that, despite fluctuations, the small molecules normally returned to their initial docking position, indicating that they could bind stably to the receptor. The visualization of the RMSD curve in the AKT1-berlambine group revealed that the protein and small molecules formed a stable complex conformation. Small molecule binding had an insignificant effect on the protein conformation. The visualization of the RMSD curve of the JUN-berlambine group indicated that the binding position of small molecules was shifted to some extent compared with the initial docking position.

Similarly, the visualization of the RMSD curve in the SRC-rhein group demonstrated that the protein and small molecules formed a stable complex conformation. Small molecule binding initially affected the protein conformation but was eventually stable after the induced fit. The conformational comparison results of the four groups before and after kinetics demonstrate that the small molecules bind stably to the receptor protein and have good binding activity, indicating that these active ingredients may contribute to the therapeutic effect of “DH-HL-JH” in WD-associated LF. However, the mechanism of high-frequency TCM against LF remains unknown.

In the present study, CHM is generally safe and well-tolerated in WD patients. However, it is still impossible to confirm the safety of using CHM because only some studies mentioned intervention safety or investigated adverse reactions. It is suggested that more effort be put into documenting and reporting these interventions' adverse effects. Simultaneously, WD is a chronic disease that requires lifelong treatment. Long-term efficacy and safety are important evaluation indicators for determining the clinical effectiveness of drug therapy. However, the duration of treatment in the present study was 32 to 336 days. The long-term safety of TCM in treating WD is unknown due to the short course of treatment. Related studies indicated that (56) treatment time is closely related to positive clinical outcomes, and it is recommended that treatment time for future trials must be longer than 1 year. There are still some inherent and methodological weaknesses in the preliminary study: (1) Due to the particularity of color, smell and taste of CHM, it is difficult to achieve blind method in clinical trials. (2) All trials were carried out in China, which may limit the generalizability. Further international multicenter RCTs of TCM for WD are needed.

## 5. Conclusion

### 5.1. Implications for practice

The combination of high-frequency CHMs may be an effective treatment for WD LF and may have potential therapeutic value for patients with various TCM syndrome types.

### 5.2. Implications for research

For patients with WD LF, there has been a gradual increase in the use of integrated traditional Chinese and Western medicine over the last few decades. The meta-analysis has shown TCM to be beneficial in treating WD LF patients and improving LF. Through network pharmacological experiments and MD simulation technology, it was identified that the three high-frequency CHMs of Rhei Radix Et Rhizoma-Coptidis Rhizoma-Curcumae Longae Rhizoma, could act on the core targets of LF, such as AKT1, SRC, and JUN through the core ingredients such as rhein, quercetin and berlambine, regulate the signal pathway and play the role of anti-LF.

## Data availability statement

The original contributions presented in the study are included in the article/Supplementary material, further inquiries can be directed to the corresponding author.

## Author contributions

PW and XY proposed and designed this study. XY, TW, YS, and YT jointly completed the data acquisition. XY and TW analyzed the data, interpreted the results, and contributed to writing the manuscript. PW and YG critically revised the manuscript for important intellectual content. All authors read and approved the final manuscript.
