# Supplementary material for: Treatment of liver fibrosis in hepatolenticular degeneration with traditional Chinese medicine: systematic review of meta-analysis, network pharmacology and molecular dynamics simulation
Source: Front Med (Lausanne). 2023 May 11;10:1193132. doi: 10.3389/fmed.2023.1193132 (PMC10213944; doi:10.3389/fmed.2023.1193132)
Supplement: Supplementary file 1 [file Data_Sheet_1.docx]

Supplementary Material

Treatment of Liver Fibrosis in Hepatolenticular Degeneration with Traditional Chinese Medicine: Systematic Review of Meta-Analysis, Network Pharmacology and Molecular Dynamics Simulation

Xulong Yang *, Tiancheng Wang *, Yiping Tang, Yawen Shao , Yaqin Gao , Peng Wu

*** Correspondence:** Peng Wu: azywupeng@ahtcm.edu.cn

# Supplementary Figures


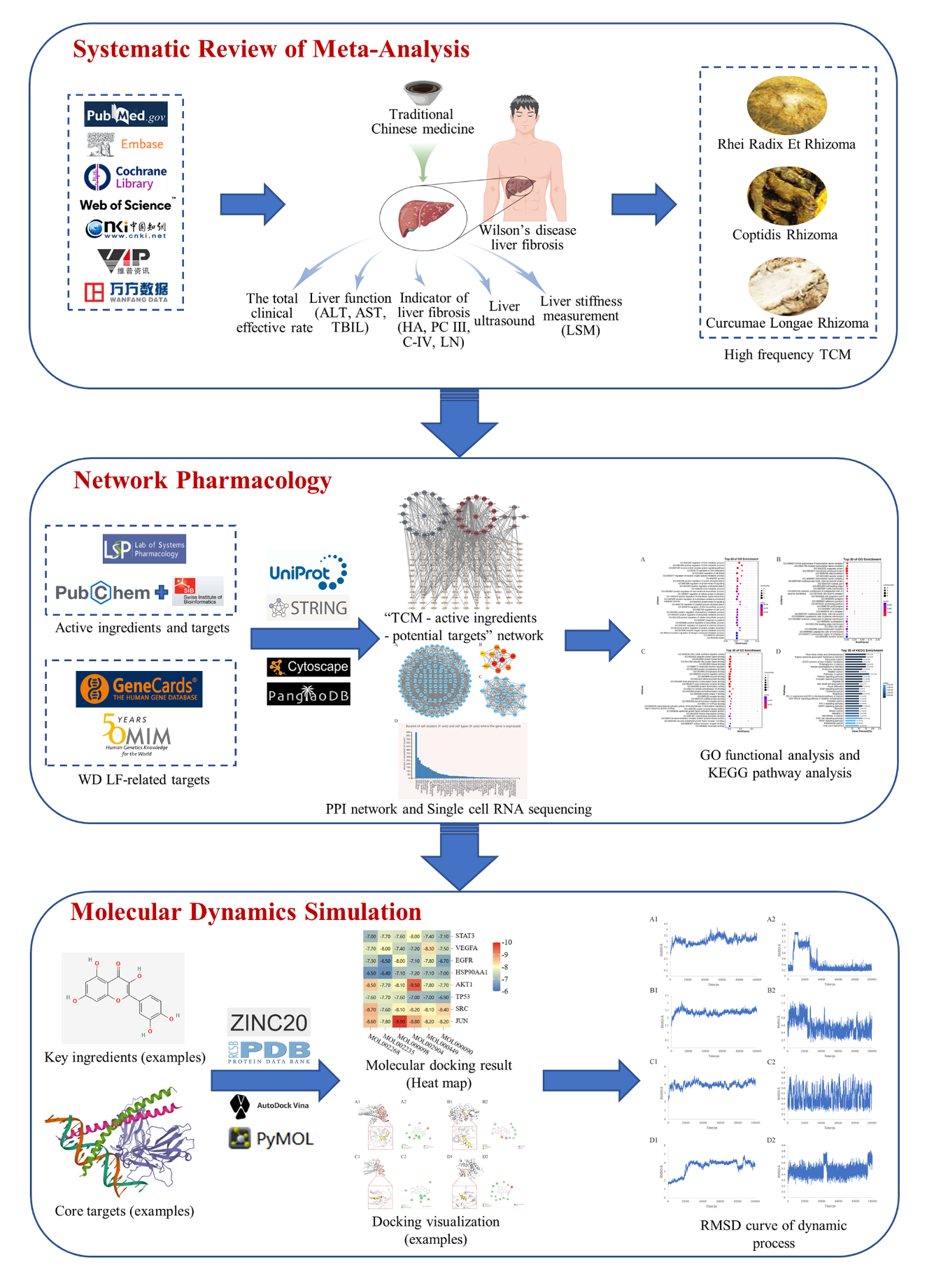


**Supplementary Figure 1.** Graphical abstract: Workflow of the investigation strategy of traditional Chinese medicine in the treatment of liver fibrosis in hepatolenticular degeneration. Three parts include systematic review of meta-analysis, network pharmacology and molecular dynamics simulation.
